# Factors Associated With Midwives' Perceived Difficulty in Supporting Couples With Older First‐Time Mothers by Workplace Type in Japan

**DOI:** 10.1111/jjns.70082

**Published:** 2026-10-09

**Authors:** Chisato Kinoshita, Mami Tamaue

**Affiliations:** ^1^ Women's Health Care Science Advanced Care Science Field Osaka Metropolitan University Graduate School of Nursing Osaka Japan

**Keywords:** child‐rearing support, midwives, older first‐time mothers, social support, workplace

## Abstract

**Aim:**

This study aimed to examine differences in midwives' perceived difficulty in providing child‐rearing and social support to couples with older first‐time mothers according to the workplace setting, and to identify contributing individual and organizational factors.

**Methods:**

A cross‐sectional survey was conducted among midwives working at medical institutions and midwifery centers across Japan who had supported couples with older first‐time mothers within the previous year. Data on individual characteristics, workplace characteristics, perceived difficulty in providing child‐rearing support, and perceived difficulty in providing social support were collected using online or anonymous self‐administered questionnaires. Workplace settings were categorized into four types: perinatal medical centers, general hospitals, clinics, and midwifery centers. Logistic regression analysis was conducted.

**Results:**

A total of 922 midwives were included. Compared with midwives working in midwifery centers, those working in perinatal medical centers (odds ratio [OR] = 2.71), general hospitals (OR = 2.67), and clinics (OR = 1.94) reported significantly greater perceived difficulty in providing child‐rearing support. Midwives with fewer than 10 years' experience also reported greater difficulty than those with 30 or more years' experience. Perceived difficulty in providing instrumental, informational, emotional, and appraisal support was associated with workplace setting. Additional associations were observed with CLoCMiP Level III certification and the availability of postpartum outreach and day‐care services.

**Conclusions:**

Midwives' perceived difficulty differed according to workplace setting and was associated with both individual and organizational factors. Strengthening organizational support systems and educational opportunities may help reduce perceived difficulty in supporting couples with older first‐time mothers.

## 1 Introduction

In recent years, however, the average age at childbirth has risen worldwide, and pregnancies at age 35 and older (Advanced Maternal Age: AMA) have increased. In Japan too, the mean age at first birth has reached 31.0 years (Ministry of Health, Labour and Welfare 2024). One contributing factor is the growing number of older first‐time mothers. Although the number of births has been declining, the proportion of births to older first‐time mothers has continued to rise: 21.2% in 2022 and 21.6% in 2023 (Portal Site of Official Statistics of Japan 2023, 2024).

AMA has been associated with higher risks of maternal complications (such as preterm birth, gestational diabetes, hypertensive disorders of pregnancy, and cesarean delivery) and fetal complications (including chromosomal abnormalities, low birth weight, and preterm birth) that correlate with maternal age (Glick et al. 2021; Mehari et al. 2020). Studies of older first‐time mothers in Japan have reported higher rates of infertility treatment, pregnancy complications, cesarean delivery, postpartum anemia, and fatigue compared to younger mothers (Mori et al. 2017). However, because older first‐time mothers in Japan are often considered medically high risk, many choose to give birth in hospitals, and a considerable number are not eligible to select midwifery centers. Therefore, it can be anticipated that hospital‐based midwives assume primary responsibility for providing child‐rearing support to older first‐time mothers.

Although mothers of advanced maternal age may possess psychosocial strengths, older first‐time mothers in Japan have also been reported to exhibit higher levels of postpartum depressive symptoms and lower maternal role confidence (Ahmad et al. 2024; Mori et al. 2017). Interventions for couples with older first‐time mothers during pregnancy that increase understanding of the physical and psychological risks borne by older mothers have suggested reductions in postpartum depression risk and improvements in couple relationships (Nakajima et al. 2023). Therefore, support for couples from pregnancy through the transition to parenthood is necessary.

As part of child‐rearing support aimed at facilitating maternal role attainment among older first‐time mothers, the provision of social support by healthcare professionals, including midwives, is also an important component (Tarkka 2003). House (1981) proposed four types of social support: instrumental, informational, emotional, and appraisal. Previous studies have reported that informational and appraisal support provided by midwives improves first‐time mothers' confidence (McLeish et al. 2021), and that emotional and appraisal support provided through public health nurse home visits is associated with higher QOL among first‐time mothers (Bernitz et al. 2016). For older first‐time mothers in Japan, satisfaction with emotional support is associated with increased maternal role confidence (Maehara et al. 2016). Thus, social support is an important form of support that facilitates adaptation and the transition to parenthood among couples with older first‐time mothers. In Japan, the Nursing Guidelines for Childrearing Support in Older Primiparas in the First Month Postpartum (NGCSOP) (Chiba University 2011) was prepared in 2014. Various difficulties have been reported in implementing the guidelines in clinical settings (Aoki et al. 2019), suggesting that midwives providing social support may experience perceived difficulty. However, the provision of social support requires opportunities for midwives to engage continuously with their clients, as well as adequate human and time resources. Therefore, workplace characteristics may influence both the provision of social support and the perceived difficulty associated with providing such support, highlighting the importance of the workplace environment.

Previous studies have primarily focused on the outcomes of couples with older first‐time mothers or on the effects of support interventions, with limited attention paid to the perspectives of midwives who provide such support. In particular, midwives' perceived difficulty in providing child‐rearing support and social support has not been sufficiently examined. Midwives' perceived difficulty may influence the quality and continuity of support, and consequently may also affect the nature and content of support provided to couples with older first‐time mothers. In Japan, a midwife's workplace refers to a delivery facility within the Japanese perinatal care system, such as perinatal medical center, general hospital, clinic, and midwifery center. Japan's perinatal care system is organized in a tiered network structure comprising primary, secondary, and tertiary levels of care. Comprehensive perinatal medical centers function as tertiary referral institutions responsible for high‐risk pregnancies and maternal‐neonatal transport, forming the core of the perinatal network system. Regional perinatal medical centers are responsible for secondary medical care areas, whereas general hospitals and clinics primarily provide primary obstetric care for low‐ to moderate‐risk pregnancies. Midwifery centers provide midwife‐led care for women with normal pregnancy courses and are required to arrange transfer to medical institutions when abnormalities are anticipated (Ministry of Health, Labour and Welfare 2000). Thus, these workplace categories differ structurally in terms of risk profile, midwives' professional discretion, continuity of care systems, and clinical roles within the national perinatal care system. However, no quantitative studies have examined the association between workplace characteristics and perceived difficulty in providing child‐rearing support and social support to couples with older first‐time mothers.

Therefore, this study aimed to examine the association between workplace setting and factors related to perceived difficulty in providing child‐rearing support and social support to couples with older first‐time mothers.

## 2 Methods

### 2.1 Study Aim

The aim of this study was to examine differences in perceived difficulty in providing child‐rearing support and social support to couples with older first‐time mothers according to the workplace setting and to identify individual and organizational factors associated with these perceived difficulties.

### 2.2 Conceptual Framework (Figure 1)

**FIGURE 1:**
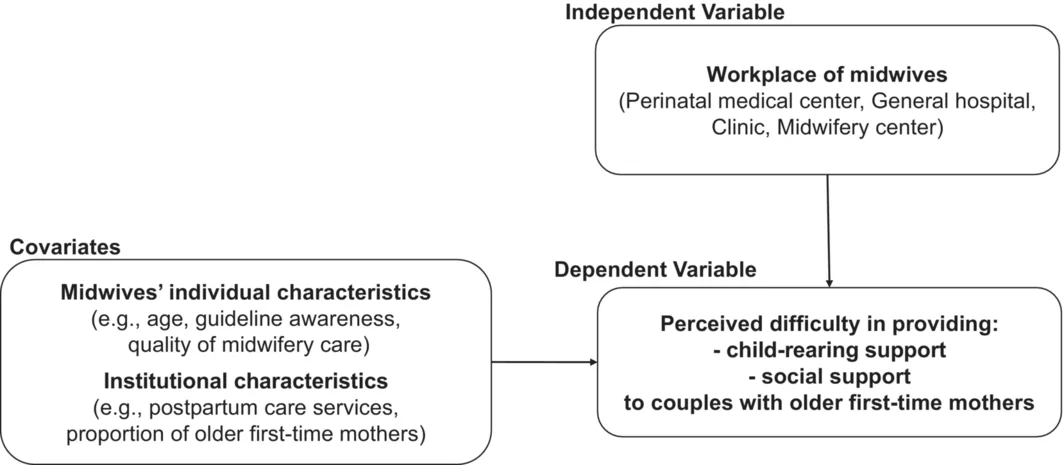
Conceptual framework.

The conceptual framework is shown in Figure 1. In the present study, the workplace of the midwives was positioned as the primary independent variable. Differences in staffing structure, models of care, role allocation, and priorities in medical management across facilities may influence midwives' scope of practice and the type of support they are able to provide. These workplace characteristics were therefore assumed to be associated with midwives' perceived difficulty in providing child‐rearing support and social support to couples with older first‐time mothers.

The dependent variable was midwives' perceived difficulty in providing child‐rearing support and social support to couples with older first‐time mothers.

In addition, the individual characteristics of the midwives (e.g., age, awareness of clinical guidelines, and self‐evaluated quality of midwifery care) and the workplace characteristics (e.g., availability of postpartum care services and the proportion of older first‐time mothers in the institution) may also influence perceived difficulty. These variables were therefore included as covariates in the analysis.

### 2.3 Operational Definitions

In this study, older first‐time mothers were defined as primiparous women aged 35 years or older at delivery, according to the definition of older primipara in the Glossary of Obstetrics and Gynecology (5th ed.) published by the Japan Society of Obstetrics and Gynecology (JSOG 2025), and consistent with the definition of advanced maternal age used in international obstetric practice (ACOG 2022).

Social support was conceptualized according to House's framework (House 1981), which comprises four types of support: instrumental, informational, emotional, and appraisal. Instrumental support was defined as direct assistance with childcare and household tasks; informational support as the provision of child‐rearing‐related information; emotional support as listening to concerns and emotions related to child‐rearing; and appraisal support as acknowledgment and affirmation of parents' efforts and adaptation.

### 2.4 Study Design

This study employed a cross‐sectional observational design targeting midwives across Japan who were involved in providing child‐rearing support to couples with older first‐time mothers.

### 2.5 Participants

The study population comprised midwives working at 2627 medical institutions (Japan Council for Quality Health Care 2024) and 799 midwifery centers (Japanese Midwives Association 2024) as of the end of January 2024. Eligible participants were midwives who had provided child‐rearing support to couples with older first‐time mothers, either during hospitalization or at outpatient clinics during the postpartum period. In medical institutions, administrators were asked to select midwives across different levels of professional experience (less than 5 years, 5–10 years, 11–20 years, and 20 years or more), with the aim of including one midwife from each experience category whenever possible to avoid overrepresentation of a particular experience group. Midwives who had not engaged in postpartum child‐rearing support within the past year (e.g., those on leave or previously in managerial positions) were excluded. However, midwives who currently held managerial positions but had provided postpartum child‐rearing support within the past year were included. Because the unit of analysis in this study was the individual midwife, proportional sampling based on institution size was not performed. Instead, four midwives were selected from each institution nationwide to reduce potential regional bias, while maintaining the feasibility of conducting a nationwide survey. In addition, institutions were asked to select midwives according to their number of years of experience, in order to minimize selection bias arising from the overrepresentation of a particular experience group within each institution.

Prior to data collection, the required sample size was estimated based on recommendations for exploratory studies and an a priori power analysis conducted using G*Power. Assuming a small effect size (0.20), a significance level of 0.05, and a statistical power of 0.80, the required sample size was estimated to be 2750 midwives working in medical institutions and 212 midwives working in midwifery centers. Assuming a response rate of 30%, the target sample size was set at 9170 midwives working in medical institutions and 710 midwives working in midwifery centers.

### 2.6 Data Collection Period

Data were collected from May to December 2024.

### 2.7 Survey Items

#### 2.7.1 Basic Attributes

Regarding midwives' individual characteristics, participants were asked about their workplace (type of institution), age, number of years of experience as a midwife, number of assisted deliveries, experience working in other departments, managerial position, certification at Level III of the Clinical Ladder of Competencies of Midwifery Practice (hereinafter, CLoCMiP; Japan Nursing Association 2013), and awareness of the NGCSOP (hereinafter, “Guidelines”; Chiba University 2011).

In addition, two subscales from the “Quality Evaluation of Midwifery Care in Hospitals: Evaluation Criteria for Self‐Assessment, Second Edition” (Japanese Nursing Association 2007, hereinafter, “Quality Evaluation”) were used: “Caring” (27 items) and “Diagnosis and Care during the Postpartum Period” (40 items) (Appendices 1 and 2). CLoCMiP recommends the use of this scale as a self‐assessment tool for evaluating midwifery competencies. This scale assesses midwives' ethical sensitivity, ability to understand clients' conditions and needs, assessment of physical, psychological, and social aspects, and provision of care that respects clients' autonomy. In this study, the “Caring” subscale was used to evaluate midwives' caregiving attitudes, and the “Diagnosis and Care during the Postpartum Period” subscale was used to assess the quality of support related to child‐rearing. Participants were asked to respond based on their practice in supporting couples with older first‐time mothers during the previous year. Permission to use and partially modify the scale was obtained from the Japan Nursing Association.

Regarding workplace characteristics, participants were asked about the prefecture in which their institution was located, as well as the length of postpartum hospitalization, duration of post‐discharge follow‐up, and availability and type of postpartum care services. Additionally, the proportion of older first‐time mothers was calculated for each prefecture using 2022 birth data.

“Workplace setting” was categorized into four types based on the Japanese perinatal care system: (1) comprehensive and regional perinatal medical centers, (2) general hospitals (≥ 20 beds providing delivery services, excluding perinatal medical centers), (3) clinics (≤ 19 beds providing delivery services), and (4) midwifery centers.

#### 2.7.2 Perceptions Regarding Child‐Rearing Support for Couples With Older First‐Time Mothers (Hereinafter, “Child‐rearing Support Difficulty”) (Appendix 3)

Perceived difficulty in providing child‐rearing support was defined as the degree to which midwives subjectively recognized difficulty when supporting couples with older first‐time mothers.

The perceived difficulty in providing child‐rearing support to couples with older first‐time mothers was assessed using a 10‐item scale developed by Isoyama and Kinugawa (2020), which focuses on understanding and perceptions related to support for family role acquisition during the perinatal period. Responses were rated on a four‐point Likert scale (1 = “Do not agree” to 4 = “Strongly agree”). The original study reported the acceptable reliability (α = 0.742). In this study, the scale was also considered suitable, and permission to use and partially modify the items was obtained from the original author.

#### 2.7.3 Perceived Difficulty in Providing Social Support to Couples With Older First‐Time Mothers (Hereinafter, “Social Support Provision Difficulty”)

Perceived difficulty in providing social support was defined as the degree to which midwives subjectively recognized difficulty in delivering these forms of support to couples with older first‐time mothers.

Participants were asked to indicate which types of social support they found difficulty providing (instrumental, informational, emotional, or appraisal). Multiple responses or the response “none” were allowed.

### 2.8 Data Collection Procedure

For medical institutions (perinatal medical centers, general hospitals, and clinics), the head of the nursing department or obstetrics unit selected four eligible midwives to participate. The midwives were informed of the study purpose, methods, and confidentiality assurances through both written and oral explanations, and were asked to complete the survey via smartphone using the Research Electronic Data Capture (hereinafter, “REDCap”) system (Harris et al. 2009, 2019).

For midwifery centers, given that 23.1% of midwives were aged 60 years or older (Japanese Midwives Association 2022), and considering the potential difficulties of online participation, anonymous self‐administered paper questionnaires were distributed.

### 2.9 Data Analysis

Participants with missing responses to the items assessing perceived difficulty in providing child‐rearing support or perceived difficulty in providing social support were excluded from the analyses. Therefore, only complete‐case data were analyzed.

Descriptive statistics were calculated for the basic attributes. Responses to the Child‐rearing Support Difficulty scale were categorized as “Difficulty present” if participants answered “Strongly agree” or “Agree”; and “Difficulty absent” if they answered “Disagree” or “Strongly disagree.” Based on the number of “Difficulty present” items among the 10 items, difficulty was classified as follows: “None” (0 items), “Weak” (1–3 items), “Moderate” (4–6 items), or “Strong” (7–10 items)—and was summarized by type of institution.

To explore factors associated with the strength of Child‐rearing Support Difficulty and the presence of Social Support Provision Difficulty, univariate logistic regression analyses were first performed. To identify factors associated with the level of Child‐rearing Support Difficulty, an ordinal logistic regression was performed with the level of Child‐rearing Support Difficulty as the dependent variable and the workplace setting as the independent variable.

For Social Support Provision Difficulty, separate binary logistic regression analyses were conducted for each of the four domains of social support, using the presence or absence of perceived difficulty in providing instrumental, informational, emotional, and appraisal support as dependent variables and workplace setting as the independent variable.

Based on theoretical relevance, and after confirming the absence of multicollinearity, years of experience, awareness of the guidelines, availability of CLoCMiP Level III, availability of postpartum short‐stay services, day‐care services, and outreach services, scores for each subscale of the Quality Evaluation and the proportion of older first‐time mothers in each prefecture were entered as covariates.

Explanatory power of the ordinal logistic regression model for the level of Child‐rearing Support Difficulty was evaluated using Nagelkerke's *R* ^2^. For the logistic regression analyses of perceived difficulty in providing social support, model fit was assessed using the Hosmer‐Lemeshow test, and explanatory power was likewise evaluated using Nagelkerke's *R* ^2^.

All statistical analyses were performed using SAS OnDemand for Academics (SAS Institute Inc.), with the significance level set at 5%.

### 2.10 Ethical Considerations

Written consent was obtained from the head of the nursing or obstetrics department at the participating medical institutions. After the consent form was submitted, four midwives were selected. Midwives provided their consent by checking an agreement box on the REDCap survey page after reading explanations regarding the study's purpose, anonymity, and participation conditions.

For midwifery centers, written explanations were provided, and midwives indicated their consent by checking a box on the questionnaire's cover page.

Research data were stored securely within the REDCap database at Osaka Metropolitan University, accessible only by the principal investigator and authorized research team members.

This study was approved by the Ethics Committee of Graduate School of Nursing, Osaka Metropolitan University (Approval No. 2024–1), May 17, 2024.

## 3 Results

Of 2627 medical institutions contacted, 87 were unreachable due to address error, cessation of delivery, or closure. We received cooperation consent forms from 353 medical institutions (response rate, 13.9%). Responses were obtained from 1090 midwives at medical institutions, of whom 786 were included in the analysis (valid response rate, 7.7%). We requested cooperation from 724 midwifery centers and received 166 responses (response rate, 22.9%), of which 136 were included in the analysis (valid response rate, 18.8%). In this study, the workplace setting was treated as the primary independent variable; therefore, the characteristics of each workplace and their associations with perceived difficulty are presented.

### 3.1 Participant Characteristics (Table 1)

**TABLE 1:** Basic attributes of the midwives overall and by type of medical institution.

	Total	Perinatal medical center	General hospital	Clinic	Midwifery center	*p*
	*n* = 922	*n* = 271	*n* = 254	*n* = 261	*n* = 136	
Age (years)	42.7 (11.0)	37.7 (9.8)	41.0 (10.3)	44.6 (10.1)	52.3 (9.6)	**< 0.001** ^**a**^
20–29	146 (15.8)	79 (29.2)	45 (17.7)	22 (8.4)	0 (0.0)	
30–39	219 (23.8)	75 (27.7)	66 (26.0)	66 (25.3)	12 (8.82)	
40–49	287 (31.1)	79 (29.2)	82 (32.3)	85 (32.6)	41 (30.2)	
50–59	206 (22.3)	36 (13.3)	54 (21.3)	69 (26.4)	47 (34.6)	
60–69	57 (6.2)	2 (0.7)	7 (2.8)	19 (7.3)	29 (21.3)	
≥ 70	7 (0.8)	0 (0.0)	0 (0.0)	0 (0.0)	7 (5.2)	
Length of midwifery experience (years)	17.1 (10.6)	13.0 (8.9)	15.1 (9.4)	18.4 (10.3)	26.3 (10.1)	**< 0.001** ^**a**^
4 ≤	98 (10.6)	52 (19.2)	30 (11.8)	16 (6.2)	0 (0.0)	
5–9	161 (17.5)	61 (22.5)	51 (20.1)	47 (18.1)	2 (1.5)	
10–14	161 (17.5)	58 (22.8)	58 (22.8)	40 (15.4)	13 (9.6)	
15–19	134 (14.5)	37 (14.6)	37 (14.6)	36 (13.9)	21 (15.4)	
20–24	134 (14.5)	29 (114)	29 (11.4)	45 (17.3)	23 (16.9)	
25–29	100 (10.8)	21 (8.3)	21 (8.3)	35 (13.5)	29 (21.3)	
≥ 30	133 (14.4)	28 (11.0)	28 (11.0)	41 (15.8)	48 (35.3)	
Number of deliveries assistances (cases)	475.7 (643.9)	253.6 (268.3)	407.8 (455.3)	674.3 (748.0)	670.6 (1011.6)	**< 0.001** ^**a**^
49 ≤	82 (8.9)	50 (18.5)	21 (8.3)	9 (3.5)	2 (1.5)	
50–99	67 (7.3)	24 (8.9)	25 (9.8)	11 (4.2)	7 (5.4)	
100–499	439 (47.6)	153 (56.7)	124 (48.8)	112 (42.9)	50 (38.5)	
500–999	203 (22.0)	30 (11.1)	62 (24.4)	71 (26.8)	41 (31.5)	
≥ 1000	124 (13.4)	13 (4.8)	22 (8.7)	59 (22.6)	30 (23.1)	
Experience in other departments						
Yes	535 (58.0)	143 (52.8)	157 (61.8)	138 (52.9)	97 (71.3)	**< 0.001** ^**b**^
No	387 (42.0)	128 (47.2)	97 (38.2)	123 (47.1)	39 (28.7)	
Managerial role						
Available	177 (19.2)	64 (23.6)	48 (18.)	65 (24.9)	—	0.229^b^
Not available	745 (80.8)	207 (76.7)	206 (81.1)	196 (75.1)	—	
Awareness of the guideline						
Yes	134 (14.5)	47 (17.3)	29 (11.4)	36 (13.8)	22 (16.2)	0.249^b^
No	788 (85.5)	224 (82.7)	225 (88.6)	225 (86.2)	114 (83.8)	
Availability of CLoCMiP Level III						
Available	339 (36.8)	117 (43.2)	90 (35.4)	79 (30.3)	53 (39.0)	**0.019** ^**b**^
Not available	583 (36.2)	154 (56.8)	164 (64.6)	182 (69.7)	83 (61.0)	
Quality evaluation of midwifery care scale in hospitals						
Caring	3.4 (0.5)	3.3 (0.5)	3.3 (0.6)	3.3 (0.5)	3.7 (0.3)	**< 0.001** ^**a**^
Diagnosis and care during the postpartum period	3.0 (0.7)	3.0 (0.7)	2.9 (0.7)	2.9 (0.7)	3.4 (0.5)	**< 0.001** ^**a**^
Length of postpartum hospitalization (days)	4.9 (1.4)	5.4 (0.8)	5.4 (0.7)	4.9 (0.8)	2.2 (2.4)	**< 0.001** ^**a**^
Duration of postpartum follow‐up (months)	3.2 (6.8)	2.4 (6.24)	2.3 (4.7)	3.4 (7.4)	9.7 (9.9)	**< 0.001** ^**a**^
Availability of postpartum care services						
Yes^c^	634 (68.8)	146 (53.9)	174 (68.5)	210 (80.5)	125 (91.9)	**< 0.001** ^**b**^
Short stay	497 (53.9)	105 (38.8)	149 (58.7)	196 (75.1)	47 (34.6)	**< 0.001** ^**b**^
Day‐care	404 (43.8)	54 (19.9)	111 (43.7)	165 (63.2)	74 (54.4)	**< 0.001** ^**b**^
Outreach	158 (17.1)	14 (5.2)	21 (8.3)	17 (6.5)	106 (77.9)	**< 0.001** ^**b**^
No	288 (31.2)	125 (46.1)	80 (31.5)	51 (19.5)	11 (8.1)	

*Note:* Age, years of midwifery experience, number of deliveries supported, quality assessment of midwifery care scale, duration of hospitalization after delivery, and postpartum follow‐up are presented as mean (standard deviation); all other variables are presented as *n* (%). A *p*‐value < 0.05 was considered statistically significant. Bold values indicate statistically significant results (*p* < 0.05).

^a^ Comparisons were performed using the Kruskal–Wallis test.

^b^ Comparisons were performed using the Chi‐squared test.

^c^ The “Yes” responses to “Availability of care services after childbirth” represent multiple responses for the services provided.

Table 1 summarizes the institution recruitment and response rates. Midwives at midwifery centers were the oldest on average and had the longest length of midwifery experience, the highest number of assisted births, and the longest postpartum follow‐up periods (all *p* < 0.001). They also had the highest mean scores on the Quality Evaluation subscale scores of “Caring” and “Diagnosis and Care during the Postpartum Period” (*p* < 0.001). Midwifery centers had the highest prevalence of postpartum care services and outreach implementation. Meanwhile, midwives working at perinatal medical centers had the lowest mean age, length of midwifery experience, and number of assisted deliveries and yet demonstrated the highest availability of CLoCMiP Level III certification.

### 3.2 Factors Associated With the Strength of Child‐Rearing Support Difficulty (Table 2)

**TABLE 2:** Factors associated with difficulty in providing childcare support: Results of an ordinal logistic regression analysis.

	*N*	Childcare support difficulty level				Multivariate analysis		
		None	Weak	Moderate	Strong	OR	95% CI	*p*
		(0)	(1–3)	(4–6)	(7–10)			
Facilities								
Perinatal medical center	271	2 (0.7)	21 (7.8)	99 (36.5)	149 (55.0)	**2.71**	1.54–4.77	**< 0.001**
General hospital	254	0 (0.0)	26 (10.2)	96 (37.8)	132 (52.0)	**2.67**	1.55–4.62	**< 0.001**
Clinic	261	2 (0.8)	40 (15.3)	107 (41.0)	112 (42.9)	**1.94**	1.13–3.36	**0.02**
Midwifery center	136	0 (0.0)	32 (23.5)	86 (63.2)	18 (13.2)	1.00		
Length of midwifery experience								
4 ≤	98	0 (0.0)	5 (5.1)	31 (31.6)	62 (63.3)	**3.38**	1.80–6.32	**< 0.001**
5–9	161	1 (0.6)	13 (8.1)	53 (32.9)	94 (58.4)	**1.83**	1.11–3.02	**0.02**
10–14	161	1 (0.6)	19 (11.8)	66 (41.0)	75 (46.6)	1.42	0.89–2.23	0.14
15–19	134	0 (0.0)	15 (11.2)	60 (44.8)	59 (44.0)	1.09	0.68–1.75	0.71
20–24	134	1 (0.8)	22 (16.4)	57 (42.5)	54 (40.3)	0.93	0.59–1.49	0.77
25–29	100	0 (0.0)	21 (21.0)	52 (52.0)	27 (27.0)	0.80	0.48–1.31	0.37
≥ 30	133	1 (0.8)	24 (18.1)	69 (51.9)	39 (29.3)	1.00		
Acknowledge guidelines								
No	788	3 (0.4)	105 (13.3)	322 (40.9)	358 (45.4)	0.79	0.55–1.14	0.21
Yes	134	1 (0.8)	14 (10.5)	66 (49.3)	53 (39.6)	1.00		
Availability of CLoCMiP Level III								
Not available	583	4 (0.7)	73 (12.5)	232 (39.8)	274 (47.0)	1.01	0.75–1.35	0.97
Available	339	0 (0.0)	46 (13.6)	156 (46.0)	137 (40.4)	1.00		
Short stay								
Not available	425	1 (0.2)	54 (12.7)	179 (42.1)	191 (44.9)	1.03	0.76–1.39	0.84
Available	497	3 (0.6)	65 (13.1)	209 (42.1)	220 (44.3)	1.00		
Day‐care								
Not available	518	2 (0.4)	66 (12.7)	217 (41.9)	233 (45.0)	0.94	0.70–1.27	0.70
Available	404	2 (0.5)	53 (13.1)	171 (42.3)	178 (44.1)	1.00		
Outreach								
Not available	764	4 (0.5)	91 (11.9)	293 (38.4)	376 (49.2)	1.40	0.89–2.20	0.15
Available	158	0 (0.0)	28 (17.7)	95 (60.1)	35 (22.2)	1.00		
Quality evaluation of midwifery care scale in hospitals								
Caring						0.92	0.70–1.22	0.57
Diagnosis and care during the postpartum period						1.14	0.89–1.45	0.30
Percentage of older first‐time mothers						1.00	0.97–1.04	0.92
Nagelkerke *R* ^2^								0.126

*Note:* A *p*‐value < 0.05 was considered statistically significant. Bold values indicate statistically significant results (*p* < 0.05). Reference categories (OR = 1.00) were defined within each variable.

Abbreviations: CI = confidence interval; OR = odds ratio.

Because midwives at midwifery centers had higher average age, length of midwifery experience, and Quality Evaluation subscale scores than those in other workplace groups, we used midwifery center midwives as the reference category in multivariate analyses. The results of factors associated with the strength of Child‐rearing Support Difficulty are shown in Table 2.

The workplace setting was significantly associated with the strength of Child‐rearing Support Difficulty. Midwives working at perinatal medical centers, general hospitals, and clinics tended to report stronger Child‐rearing Support Difficulty than those working at midwifery centers (odds ratio [OR]: perinatal medical center, 2.71; 95% CI, 1.54–4.77; *p* < 0.001; general hospital, 2.67; 95% CI, 1.55–4.62; *p* < 0.001; clinic, 1.94; 95% CI, 1.13–3.36; *p* = 0.02). Midwives with less than 10 years of midwifery experience reported significantly greater difficulty compared with those with 30+ years (OR: < 4 years, 3.38; 95% CI, 1.80–6.32; *p* < 0.001; 5–9 years, 1.83; 95% CI, 1.11–3.02; *p* = 0.02).

Other factors (availability of CLoCMiP Level III, awareness of the Guidelines, Quality Evaluation, type of postpartum care services, percentage of older first‐time mothers) were not significantly associated.

The Nagelkerke *R* ^2^ value for this model was 0.126, suggesting limited explanatory power for the dependent variable.

### 3.3 Factors Associated With Each of the Four Types of Social Support Provision Difficulty (Table 3)

**TABLE 3:** Factors associated with difficulty in providing each of the four types of social support (instrumental, informational, emotional, appraisal support): Results of binary logistic regression analyses.

	N	Instrumental support				Informational support				Emotional support				Appraisal support			
		Presence of difficult to provide instrumental support	Multivariate analysis			Presence of difficult to provide informational support	Multivariate analysis			Presence of difficult to provide emotional support	Multivariate analysis			Presence of difficult to provide appraisal support	Multivariate analysis		
		Yes	OR	95% CI	*p*	Yes	OR	95% CI	*p*	Yes	OR	95% CI	*p*	Yes	OR	95% CI	*p*
Facilities																	
Perinatal medical center	271	114 (42.1)	**3.49**	1.79–6.81	**< 0.001**	81 (29.9)	**2.51**	1.13–5.18	**0.01**	118 (43.5)	**2.28**	1.22–4.28	**0.01**	106 (39.1)	**3.92**	1.88–8.17	**< 0.001**
General hospital	254	97 (38.2)	**3.24**	1.69–6.22	**< 0.001**	88 (34.7)	**3.34**	1.65–6.74	**< 0.001**	136 (53.5)	**3.23**	1.76–5.95	**< 0.001**	118 (46.5)	**5.14**	2.52–10.49	**< 0.001**
Clinic	261	86 (33.0)	**2.75**	1.42–5.32	**0.003**	60 (23.0)	**2.13**	1.03–4.37	**0.040**	113 (43.3)	**2.01**	1.09–3.72	**0.036**	98 (37.6)	**3.37**	1.64–6.93	**< 0.001**
Midwifery center	136	29 (21.3)	1.00			23 (16.9)	1.00			35 (25.7)	1.00			16 (11.8)	1.00		
Length of midwifery experience																	
4 ≤	98	40 (40.8)	1.37	0.71–2.64	0.342	36 (36.7)	1.70	0.84–3.43	0.141	49 (50.0)	1.16	0.62–2.20	0.639	43 (43.9)	1.05	0.54–2.02	0.897
5–9	161	63 (39.1)	1.31	0.75–2.28	0.340	47 (29.2)	1.35	0.73–2.49	0.343	72 (44.7)	1.00	0.59–1.71	0.995	66 (41.0)	1.01	0.57–1.77	0.981
10–14	161	54 (33.5)	0.99	0.58–1.67	0.956	51 (31.7)	1.62	0.91–2.88	0.100	76 (47.2)	1.25	0.75–2.07	0.397	61 (37.9)	1.04	0.61–1.78	0.887
15–19	134	48 (35.8)	1.10	0.64–1.87	0.737	39 (29.1)	1.47	0.82–2.65	0.196	62 (46.3)	1.32	0.79–2.21	0.292	45 (33.6)	0.94	0.54–1.64	0.826
20–24	134	43 (32.1)	0.95	0.55–1.62	0.842	24 (17.9)	0.81	0.43–1.52	0.504	61 (45.5)	1.35	0.81–2.24	0.252	49 (36.6)	1.15	0.67–1.99	0.608
25–29	100	36 (36.0)	1.32	0.75–2.32	0.342	27 (27.0)	1.61	0.86–3.01	0.138	34 (34.0)	0.93	0.53–1.64	0.813	36 (36.0)	1.41	0.78–2.54	0.259
≥ 30	133	41 (30.8)	1.00			27 (20.3)	1.00			47 (35.3)	1.00			37 (27.8)	1.00		
Acknowledge guidelines																	
No	788	279 (35.4)	1.02	0.68–1.53	0.910	221 (28.1)	1.11	0.71–1.75	0.653	347 (44.0)	0.997	0.67–1.48	0.990	292 (37.1)	1.02	0.68–1.55	0.911
Yes	134	47 (35.1)	1.00			31 (23.1)	1.00			55 (41.0)	1.00			46 (34.3)	1.00		
Availability of CLoCMiP Level III																	
Not available	583	193 (33.1)	**0.71**	0.51–0.99	**0.040**	158 (27.1)	0.80	0.56–1.15	0.232	260 (44.6)	1.07	0.78–1.47	0.694	220 (37.7)	1.03	0.74–1.44	0.846
Available	339	133 (39.2)	1.00			94 (27.7)	1.00			142 (41.9)	1.00			118 (34.8)	1.00		
Short stay																	
Not available	425	158 (37.2)	1.14	0.82–1.59	0.424	125 (29.4)	1.26	0.89–1.79	0.198	181 (42.6)	1.19	0.87–1.64	0.282	154 (36.2)	1.22	0.88–1.70	0.230
Available	497	168 (33.8)	1.00			127 (25.6)	1.00			221 (44.5)	1.00			184 (37.0)	1.00		
Day‐care																	
Not available	518	198 (38.2)	1.14	0.81–1.59	0.451	154 (29.7)	1.14	0.80–1.64	0.475	215 (41.5)	**0.68**	0.50–0.95	**0.022**	189 (36.5)	0.77	0.55–1.08	0.129
Available	404	128 (31.7)	1.00			98 (24.3)	1.00			187 (46.3)	1.00			149 (36.9)	1.00		
Outreach																	
Not available	764	276 (36.1)	0.60	0.36–1.00	0.051	211 (27.6)	**0.54**	0.31–0.93	**0.025**	349 (45.7)	0.95	0.58–1.57	0.849	309 (40.5)	1.41	0.82–2.44	0.214
Available	158	50 (31.7)	1.00			41 (26.0)	1.00			53 (33.5)	1.00			29 (18.4)	1.00		
Quality evaluation of midwifery care scale in hospitals																	
Caring			1.02	0.75–1.38	0.900		0.86	0.63–1.19	0.368		0.95	0.71–1.27	0.707		0.89	0.66–1.19	0.423
Diagnosis and care during the postpartum period			1.02	0.78–1.32	0.903		0.86	0.65–1.14	0.291		0.84	0.65–1.08	0.173		0.85	0.66–1.11	0.226
Percentage of older first‐time mothers			0.99	0.96–1.03	0.728		1.00	0.96–1.05	0.852		0.99	0.95–1.02	0.464		1.00	0.96–1.04	0.940
Significance of the Hosmer–Lemeshow Test					0.72				0.40			0.16					0.94
Nagelkerke *R* ^2^					0.05				0.06			0.06					0.09

*Note:* Binary logistic regression was used. Model was adjusted for facility type, length of midwifery experience, awareness of guidelines, availability of CLoCMiP Level III, availability of short stay and day‐care programs, outreach services, quality of midwifery care (caring, postpartum diagnosis and care), and the percentage of older first‐time mothers. A *p*‐value < 0.05 was considered statistically significant. Bold values indicate statistically significant results (*p* < 0.05). Reference categories (OR = 1.00) were defined within each variable.

Abbreviations: CI = confidence interval; OR = odds ratio.

We used midwifery center midwives as the reference category in the multivariate analysis for each of the four types of Social Support Provision Difficulty (“Yes” vs. “No”). The results are shown in Table 3.

Instrumental Support Provision Difficulty was significantly associated with workplace setting and availability of CLoCMiP Level III certification. Midwives working at perinatal medical centers, general hospitals, and clinics tended to report greater perceived difficulty in providing instrumental support than those working at midwifery centers (OR = perinatal medical center, 3.49; 95% CI, 1.79–6.81; *p* < 0.001; general hospital, 3.24; 95% CI, 1.69–6.22; *p* < 0.001; clinic, 2.75; 95% CI, 1.42–5.32; *p* = 0.003). Midwives without CLoCMiP Level III certification tended to report lower perceived difficulty in providing instrumental support than those with certification (OR = 0.71; 95% CI, 0.51–0.99; *p* = 0.040).

Informational Support Provision Difficulty was significantly associated with workplace setting and the availability of postpartum outreach services. Midwives working at perinatal medical centers, general hospitals, and clinics tended to report greater difficulty in providing informational support than those working at midwifery centers (OR = perinatal medical center, 2.51; 95% CI, 1.13–5.18; *p* = 0.010; general hospital, 3.34; 95% CI, 1.65–6.74; *p* < 0.001; clinic, 2.13; 95% CI, 1.03–4.37; *p* = 0.040). Midwives working in institutions without postpartum outreach services tended to report lower perceived difficulty in providing informational support than those working in facilities offering outreach services (OR = 0.54; 95% CI, 0.31–0.93; *p* = 0.025).

Emotional Support Provision Difficulty was significantly associated with workplace setting and the availability of postpartum day‐care services. Midwives working at perinatal medical centers, general hospitals, and clinics tended to report greater perceived difficulty in providing emotional support than those working at midwifery centers (OR = perinatal medical center, 2.28; 95% CI, 1.22–4.28; *p* = 0.010; general hospital, 3.23; 95% CI, 1.76–5.95; *p* < 0.001; clinic, 2.01; 95% CI, 1.09–3.72; *p* = 0.036). Midwives working in facilities without postpartum day‐care services tended to report lower perceived difficulty in providing emotional support than those working in facilities offering day‐care services (OR = 0.68; 95% CI, 0.50–0.95; *p* = 0.022).

Appraisal Support Provision Difficulty was significantly associated with workplace setting. Midwives working at perinatal medical centers, general hospitals, and clinics tended to report greater perceived difficulty in providing appraisal support than those working at midwifery centers (OR = perinatal medical center, 3.92; 95% CI, 1.88–8.17; *p* < 0.001; general hospital, 5.14; 95% CI, 2.52–10.49; *p* < 0.001; clinic, 3.37; 95% CI, 1.64–6.93; *p* < 0.001).

The Hosmer–Lemeshow test evaluates the discrepancy between observed and predicted probabilities, with a nonsignificant result indicating an adequate model fit. The Nagelkerke pseudo *R* ^2^ represents the explanatory power of a logistic regression model, suggesting that the model demonstrated a modest level of explanatory capacity. In each model, the Hosmer–Lemeshow test yielded *p* values ranging from 0.16 to 0.94, indicating no evidence of poor model fit. In contrast, the Nagelkerke *R* ^2^ values ranged from 0.05 to 0.09, suggesting limited explanatory power of the models for the dependent variables.

### 3.4 Comparison of Quality Evaluation Subscale Scores According to CLoCMiP Level III Certification (Supplementary Analysis)

The Quality Evaluation subscale scores were compared according to CLoCMiP Level III certification using the Mann–Whitney U test (significance level < 5%). The mean score for Caring was 3.46 in the group with CLoCMiP Level III certification and 3.31 in the group without CLoCMiP Level III certification, showing a significant difference (*z* = 4.302; *p* < 0.001).

Similarly, the mean score for Diagnosis and Care during the Postpartum Period was 3.26 in the group with CLoCMiP Level III certification and 2.84 in the group without CLoCMiP Level III certification, also showing a significant difference (*z* = 9.502; *p* < 0.001).

In both outcomes, higher scores were observed in the group with available CLoCMiP Level III certification.

## 4 Discussion

### 4.1 Factors Associated With Midwives' Perceived Difficulty in Supporting Couples With Older First‐Time Mothers According to the Midwives' Workplace

#### 4.1.1 Differences in Institutional Functions

In this study, midwives working at perinatal medical centers, general hospitals, and clinics reported greater perceived difficulty in providing child‐rearing support and each of the four types of social support to couples with older first‐time mothers compared to those working at midwifery centers. The Japanese perinatal care system has a hierarchical network structure, with comprehensive and regional perinatal medical centers serving as tertiary care facilities, and general hospitals, clinics, and midwifery centers providing primary and secondary care (Ministry of Health, Labour and Welfare 2000). Considering the functions of institutions within this system structure, structural differences are likely to exist in the risk levels of the women served, the continuity of care, and the availability of time for providing support.

Perinatal medical centers primarily manage high‐risk pregnancies and deliveries, and place emphasis on acute and advanced medical care. Consequently, securing sufficient time for child‐rearing support and social support that addresses psychosocial aspects may be challenging. In contrast, midwifery centers care for women with low‐risk pregnancies and provide continuous care from pregnancy through the postpartum period. This continuity likely facilitates relationship building and may have contributed to lower perceived difficulty in providing support.

Therefore, the association observed in this study between workplace setting and perceived difficulty can be interpreted as reflecting differences in medical function and care model. However, because this study did not directly measure the risk level of the women served, careful interpretation is required regarding the extent to which differences in perceived difficulty reflect variations in case mix.

#### 4.1.2 Years of Experience and Institutional Function

Midwives with fewer than 10 years of experience reported greater perceived difficulty in providing child‐rearing support to couples with older first‐time mothers. Barriers to building relationships between nurses and older patients include patient age, time constraints, preconceptions held by nurses or patients, and insufficient knowledge among nurses (Amoah et al. 2019; Shaban et al. 2024). In particular, perinatal medical centers and general hospitals tend to employ a higher proportion of younger midwives, while age gaps and differences in social background between younger midwives and couples with older first‐time mothers may contribute to communication‐related difficulties.

Older first‐time mothers have been reported to seek child‐rearing support while simultaneously experiencing conflicts related to age‐based self‐esteem and pride (Mori et al. 2014). Understanding the complex needs of older first‐time mothers requires advanced clinical competence and relationship‐building skills. Among less‐experienced midwives, these competencies may still be developing, thereby increasing perceived difficulty in providing support. Reflection in clinical practice has been reported as an effective strategy for enhancing nurses' competencies (Kim et al. 2018; Pai 2015). Accordingly, particularly in institutions with acute‐care functions, the establishment of mentoring systems and the provision of reflective education for early‐career midwives are essential.

These findings suggest that in medical institutions with a high proportion of younger midwives, institutional function and number of years of experience may interact to amplify perceived difficulty.

#### 4.1.3 Awareness of the Guidelines

More than 85% of participants reported being unaware of the Guidelines, and awareness of the Guidelines was not associated with either type of perceived difficulty. The Guidelines specify care to promote physical recovery in older first‐time mothers and provide concrete recommendations for concurrently addressing newborn care, breastfeeding support, and prevention of postpartum depression. However, globally, various internal and external barriers to implementing clinical practice guidelines for nursing professionals have been identified, including resource shortages, lack of awareness and knowledge, and insufficient training (Jun et al. 2016; Zhou et al. 2023).

Chen et al. (2020) suggested that continuous training for nursing staff is necessary to ensure that guidelines are effectively implemented in daily practice and that establishing mentoring roles within training programs can promote advanced nursing practice. In the present study, awareness of the Guidelines was not associated with either type of perceived difficulty. This finding may indicate that the Guidelines have not yet been sufficiently implemented in practice, which is consistent with Aoki et al. (2019), who also reported challenges in applying the Guidelines in clinical settings. Therefore, further dissemination and effective implementation of the Guidelines may help reduce perceived difficulty among midwives. These findings suggest that greater dissemination and active utilization of the Guidelines may contribute to strengthening support for older first‐time mothers in medical institutions.

To ensure successful implementation, medical institutions must promote awareness of the Guidelines, allocate appropriate personnel and resources, review workload structures, and provide continuous education and training. Through these organizational efforts, concrete support for couples with older first‐time mothers can be systematically strengthened.

#### 4.1.4 Type of Postpartum Care Service

Facilities without day‐care services showed lower perceived difficulty in providing emotional support. Savory et al. (2022) reported that midwives perceive difficulties in providing mental health support to women because time constraints force them to focus on physical care and practical childcare skills, resulting in limited time for psychological care. These findings suggest that midwives in facilities offering day‐care services may be more likely to perceive difficulties in providing emotional support.

In addition, facilities without outreach services showed lower perceived difficulty in providing informational support. Home‐based postpartum care includes various components, such as social and emotional support and empowerment, health education, assessment of the home environment, and promotion of parent–child relationships (Mespreuve et al. 2024), requiring midwives to provide multiple types of care during a single visit. Consequently, it may be difficult to allocate sufficient time and attention to informational support during each outreach visit. These factors may explain why midwives in facilities providing outreach services are more likely to perceive difficulties in delivering informational support.

#### 4.1.5 Association With Midwives' Core Competencies

Midwives without CLoCMiP Level III certification were found to report lower perceived difficulty in providing instrumental support. The “Core Competencies for Midwives” outline essential practice abilities, including ethical competence—such as respecting and advocating for the life, dignity, and rights of women, children, and families—and supporting informed decision‐making while respecting women's autonomy. In addition, maternity care competencies include providing necessary information regarding pregnancy, childbirth, and child‐rearing; supporting women's decision‐making; and facilitating reflection on pregnancy and birth experiences according to the mother's situation (Japan Midwives Association 2021). Midwives are expected to integrate these competencies in their health support activities for women. Notarnicola et al. (2023) reported that nurses' competency levels are positively correlated with years of clinical experience. A supplementary analysis showed that midwives with CLoCMiP Level III certification had higher scores for Quality Evaluation subscales, suggesting that certified midwives may possess a higher level of competency. However, the Quality Evaluation subscale score itself was not associated with perceived difficulty in providing instrumental support. Rouhi et al. (2026) identified shortages of personnel and support resources, as well as time constraints, as challenges in postpartum support services. Therefore, although midwives with CLoCMiP Level III certification may aspire to provide a higher standard of instrumental support, such as breastfeeding and childcare support, they may also be more aware of organizational constraints, which could contribute to greater perceived difficulty in providing instrumental support. In healthcare facilities where midwives are responsible for caring for multiple mothers and infants within a single day, improving midwifery staffing and support resources remains an important challenge regardless of the availability of CLoCMiP Level III certification.

### 4.2 Contribution of the Study

Because older first‐time mothers are medically high risk, births at perinatal medical centers and general hospitals are expected to increase, and midwives working in medical institutions must be prepared to support older first‐time mothers and their partners. Midwives in medical institutions should understand older first‐time mothers' social support needs, utilizing reflection and communication training. Building follow‐up systems for less experienced midwives and promoting dissemination of the Guidelines and ongoing training may improve the quality of support for older first‐time mothers and their partners. Furthermore, midwives working at midwifery centers can leverage the advantage of longer postpartum follow‐up periods to strengthen collaboration with medical institutions in postpartum care services, thereby facilitating continuous support.

### 4.3 Limitations and Future Directions

This study has several limitations. First, because participants were asked to retrospectively evaluate their care for couples with older first‐time mothers, recall bias and bias associated with subjective evaluation may have occurred. Second, although midwives from across Japan were recruited, the valid response rate was low (7%–18%), which may limit the generalizability of the findings. Furthermore, because midwives working in medical institutions were selected by their managers, midwives with a particular interest in supporting couples with older first‐time mothers may have been overrepresented, resulting in potential selection bias. In addition, because the number of participants was limited to four per institution, differences in institution size may not have been adequately reflected.

Furthermore, because information identifying individual facilities was not collected, it was not possible to account for within‐institution correlations among midwives' responses. As a result, the standard errors of some estimates may have been underestimated. Relatedly, the proportional odds assumption underlying the ordinal logistic regression model used for Child‐rearing Support Difficulty was not formally tested; violation of this assumption, if present, could affect the interpretation of the ORs obtained. In addition, both Quality Evaluation subscale scores and Child‐rearing Support Difficulty and Social Support Provision Difficulty were assessed using self‐reported questionnaires. Therefore, the possibility of common method bias cannot be excluded, and some associations may have been overestimated or underestimated. Furthermore, because this study employed a cross‐sectional design, causal relationships cannot be inferred, and the possibility of reverse causality cannot be ruled out.

In addition, comparisons with support‐related difficulties among couples experiencing their first childbirth in which the mother was younger than 35 were not conducted, and the reasons underlying midwives' perceived difficulties were not explored. Therefore, it cannot be concluded that the findings are unique to couples with older first‐time mothers. Finally, the explanatory power of the ordinal logistic regression model for the level of Child‐rearing Support Difficulty, as well as the explanatory power of the multivariable models for the four types of Social Support Provision Difficulty, was limited, suggesting that factors not considered in this study—such as the risk level of women receiving care, the number of midwives employed, and whether maternity care was provided in dedicated or mixed wards—may also have influenced the results.

Nevertheless, many participating midwives reported perceiving difficulty in supporting couples with older first‐time mothers, and identifying related factors provides meaningful insights into the issues that should be addressed within different workplace settings. Future research should clarify the reasons for perceived difficulty across workplace types and explore interventions to address them.

## 5 Conclusion

This study examined workplace differences in midwives' perceived difficulty in providing child‐rearing support and social support to couples with older first‐time mothers and explored associated individual and organizational factors.

Midwives working in perinatal medical centers, general hospitals, and clinics reported greater perceived difficulty than those working in midwifery centers, demonstrating clear workplace‐related differences. In addition, fewer years of experience and CLoCMiP Level III certification were associated with perceived difficulty as individual factors, whereas the availability and type of postpartum care services were associated with perceived difficulty as organizational factors. These findings suggest that midwives' perceived difficulty in supporting couples with older first‐time mothers may be influenced not only by individual experience and professional qualifications but also by workplace‐related organizational resources and care systems.

Although the study largely achieved its objective of clarifying differences between workplaces and associated factors, further research considering women's risk levels is needed.

## Author Contributions

C.K. contributed to the conception and design of this study, performed the statistical analysis, carried out the data collection and analysis, drafted the manuscript; C.K. and M.T. reviewed the manuscript; C.K. and M.T. contributed to the project administration of this study; M.T. critically reviewed the manuscript and supervised the whole study process. All authors read and approved the final manuscript.

## Ethics Statement

This study was approved by the Ethics Committee of Graduate School of Nursing, Osaka Metropolitan University (Approval No. 2024–1), May 17, 2024.

## Conflicts of Interest

The authors declare no conflicts of interest.

## Data Availability

The data supporting the findings of this study are not publicly available because of ethical and privacy considerations.

## Appendix 1 The Evaluation Criteria for the Quality of Midwifery Care in Medical Institutions: For Self‐Assessment, 2nd Edition “Caring”

**Table:**

1. Knowing – Striving to Understand the Person as Their Family Would
1.1 Avoids interpreting the person through assumptions or preconceptions.
1.2 Centers care around the care recipient.
1.3 Assesses the care recipient's lived experiences:
1.3.1 Conducts thorough assessments.
1.3.2 Continuously seeks cues over time.
1.4 Intentionally recognizes the person as someone important.
1.5 Values the responses expressed by the person.
2. Being With – Remaining Emotionally Present Like Their Family
2.1 Stays physically present with the person.
2.2 Is ready to respond promptly when needed.
2.3 Shares emotions with the person.
2.4 Is present in a non‐burdensome way for the person.
3. Acting for the benefit of others: Engaging in Actions for Others as Much as One Would for Oneself
3.1 Alleviates the person's suffering and sorrow.
3.2 Provides comfort.
3.3 Anticipates the person's needs.
3.4 Acts with confidence and skill.
3.5 Protects the person.
3.6 Preserves dignity by acting discreetly and without imposition.
4. Enabling – Supporting the Person's Ability to Move Forward Comfortably
4.1 Uses professional knowledge to enhance others' well‐being.
4.2 Accepts and validates the person's emotions to provide psychological support.
4.3 Provides clear information and explanations.
4.4 Focuses on what the person values and supports their thinking.
4.5 Offers accurate information and constructive feedback.
5. Maintaining Belief – Sustaining Confidence in the Person's Capabilities
5.1 Continues to believe in the capabilities of the woman and her family.
5.2 Maintains the person's self‐esteem.
5.3 Sustains a hopeful attitude.
5.4 Interacts in ways that convey realistic optimism.
5.5 Takes a long‐term perspective.
Caring Evaluation Criteria
Level 1: Is able to engage with awareness of caring when given guidance.
Level 2: Makes efforts to engage with awareness of caring.
Level 3: Generally engages with awareness of caring.
Level 4: Consistently engages with full awareness of caring.
Level 0: Does not meet the facility's minimum standard for caring.

## Appendix 2 The Evaluation Criteria for the Quality of Midwifery Care in Medical Institutions: For Self‐Assessment, 2nd Edition “Diagnosis and Care During the Postpartum Period”

**Table:**

1. Understanding and Implementation of Postpartum Care Policies
1.1 Understands the facility's policy on postpartum care for mothers and families.
1.2 Explains the postpartum care policy to mothers and families.
1.3 Provides care in accordance with the facility's policy.
2. Application of Institutional Standards and Procedures Related to Postpartum Care
2.1 Provides care based on institutional standards and procedures.
3. Assessment of Mothers' Needs
3.1 Provides information regarding normal postpartum recovery.
3.2 Assesses mothers' self‐care abilities.
3.3 Respects the autonomy of mothers.
3.4 Identifies latent needs of mothers and families.
4. Health Assessment of Postpartum Mothers
4.1 Performs postpartum assessments using appropriate methods and techniques.
4.2 Conducts accurate assessments through interviews, inspection, palpation, auscultation, and observation.
4.3 Distinguishes between normal and abnormal postpartum recovery.
4.3.1 Assesses physical recovery.
4.3.2 Assesses psychosocial aspects (e.g., maternal role attainment, family role adjustment).
4.4 Identifies or predicts abnormalities during or after postpartum recovery.
4.5 Identifies signs of mother–infant bonding disorder or high‐risk factors for child abuse.
4.6 Provides satisfactory responses to the needs of mothers and families.
5. Care Planning for Postpartum Mothers
5.1 Develops care plans in accordance with postpartum recovery.
5.1.1 Plans to promote physiological involution.
5.1.2 Plans to establish breastfeeding.
5.1.3 Plans to support mothers who are unable to or choose not to breastfeed.
5.1.4 Plans to promote bonding and maternal role attainment.
5.2 Develops individualized care plans.
5.3 Respects mothers' autonomy when creating care plans.
5.4 Modifies care plans in response to postpartum progress and emerging needs.
6. Implementation of Care Plans, Health Education, and Consultation
6.1 Plans group guidance on postpartum life, family planning, and childcare.
6.2 Conducts and manages group guidance on postpartum life, family planning, and childcare.
6.3 Implements breastfeeding support plans.
6.3.1 Provides care and guidance on frequent breastfeeding.
6.3.2 Selects appropriate feeding methods (e.g., direct breastfeeding, pumping, assistive tools, holding positions) and provides care and guidance.
6.3.3 Guides mothers in recognizing infant hunger cues.
6.3.4 Instructs mothers on techniques for maintaining lactation.
6.4 Provides individualized postpartum care and consultation regarding daily life, family planning, and childcare.
7. Evaluation of Care
7.1 Demonstrates a reflective attitude toward one's care practices.
7.2 Observes mothers' and families' responses to assess the appropriateness of care.
7.3 Confirms care effectiveness using objective indicators.
7.4 Uses evaluation results to improve care quality.
8. Care Coordination and Continuity
8.1 Selects appropriate timing and methods (e.g., documentation, verbal reporting) to initiate care coordination.
8.2 Communicates in a way that supports continued autonomy and self‐determination of mothers.
8.3 Identifies and selects appropriate professionals (e.g., medical team members, institutions, community resources) for collaboration.
8.4 Establishes care continuity and coordination that satisfies mothers and families.
Postpartum Assessment and Care Evaluation Criteria
Level 1: Provides care with guidance.
Level 2: Independently provides care.
Level 3: Provides individualized care using diverse methods to achieve specific goals.
Level 4: Accurately identifies conditions based on minimal cues and provides highly targeted care.
Level 0: Does not meet the facility's standard for care provision.

## Appendix 3 Perceived Difficulties in Providing Child‐Rearing Support to Couples With Older First‐Time Mothers

**Table:**

1. The needs of couples with older first‐time mothers are not clearly understood.
2. The specifics of care for couples with older first‐time mothers remain unclear.
3. Integrating new practices is challenging.
4. I am unable to provide care for couples with older first‐time mothers due to time constraints.
5. I recognize the need for care for couples with older first‐time mothers.
6. I have not deeply considered the care needs of couples with older first‐time mothers.
7. There is a challenge in determining the extent to which healthcare professionals can engage with couples with older first‐time mothers.
8. While care for couples with older first‐time mothers during pregnancy is important, it is currently difficult to implement.
9. An educational support system that includes couples with older first‐time mothers is necessary.
10. I would like to incorporate more support for couples with older first‐time mothers.
